# Associations between the burdens of comorbid sleep problems, central sensitization, and headache-related disability in patients with migraine

**DOI:** 10.3389/fneur.2024.1373574

**Published:** 2024-02-26

**Authors:** Keisuke Suzuki, Shiho Suzuki, Yasuo Haruyama, Kei Funakoshi, Hiroaki Fujita, Hirotaka Sakuramoto, Mai Hamaguchi, Gen Kobashi, Koichi Hirata

**Affiliations:** ^1^Department of Neurology, Dokkyo Medical University, Mibu, Japan; ^2^Integrated Research Faculty for Advanced Medical Sciences, Dokkyo Medical University, Mibu, Japan; ^3^Department of Public Health, Dokkyo Medical University, Mibu, Japan

**Keywords:** migraine, central sensitization, sleep disorders, headache-related disability, insomnia

## Abstract

**Objective:**

Sleep disturbances are common in migraine patients and affect quality of life. Central sensitization (CS) is likely to play a role in the increased severity and chronicity of migraine. We hypothesized that the number of comorbid sleep problems would affect headache-related disability through the effects of central sensitization (CS).

**Methods:**

We performed a cross-sectional study including 215 consecutive patients with migraine. Insomnia was defined as a Pittsburgh Sleep Quality Index (PSQI) global score greater than 5. Probable REM sleep behavior disorder (pRBD) was defined as an RBD screening score of 5 or greater. Excessive daytime sleepiness (EDS) was defined as an Epworth Sleepiness Scale score of 10 or higher. Suspected sleep apnea (SA) was defined as patients with snoring or sleep apnea witnessed 3 or more nights a week. CS was assessed by the Central Sensitization Inventory (CSI).

**Results:**

Restless legs syndrome, insomnia, EDS, SA and pRBD were observed in 25.6%, 71.6%, 34.4%, 10.2%, and 21.4%, respectively, of the patients. At least one sleep problem was present in 87.0% of the patients. According to the results of the multinomial logistic regression analysis with no sleep problems as a reference, after we corrected for adjustment factors, the Migraine Disability Assessment (MIDAS) score significantly increased when three or more comorbid sleep problems were present. According to our mediation analysis, an increased number of sleep problems had a direct effect on the MIDAS score after we adjusted for other variables, and the CSI score was indirectly involved in this association.

**Conclusion:**

The present study showed an association between migraine-related disability and the burden of multiple sleep problems, which was partially mediated by CS.

## Introduction

Migraine is a neurological disorder characterized by moderate or severe headache attacks and reversible neurological and systemic symptoms and is accompanied by photophobia, phonophobia, cutaneous allodynia, nausea and vomiting (1). The pathophysiology of migraine includes the release of neuropeptides, such as calcitonin gene-related peptide (CGRP), from trigeminal nerve endings, neurogenic inflammation, activation of the trigeminal vascular systems, and hypothalamic activation, which is associated with an altered sleep–wake cycle, yawning and food cravings (2).

Sleep disorders are common in patients with migraine and include insomnia (difficulty falling asleep, difficulty staying asleep and early morning awakenings), excessive daytime sleepiness (EDS), restless legs syndrome (RLS), rapid eye movement (REM) or non-REM sleep parasomnia, narcolepsy and obstructive sleep apnea (OSA) (3–6). Patients with chronic migraine, which manifests as 15 or more headache days per month, have more sleep problems that negatively impact their daily life than those with episodic migraine, manifesting as 14 or fewer headache days per month (7, 8). Insomnia is a common sleep complaint reported by half to two-thirds of patients with migraine (5), and its comorbidity rate is increased in chronic migraine patients with severe, nocturnal, and/or awakening headache patterns (4). Habitual snoring, a major symptom of obstructive sleep apnea, has been reported to be a risk factor for chronic migraine (9). A meta-analysis of 15,402 OSA patients and 23 related studies revealed a pooled prevalence of all headaches in patients with OSA of 33%, sleep apnea headache of 25%, tension-type headache of 19%, and migraine of 16%. Although OSA did not significantly increase the risk of headache occurrence compared to non-OSA in that study, the findings underscore the importance of screening for headache in patients with sleep disorders and screening for sleep disorders in patients with headache (10). Recent evidence suggests that a bidirectional relationship exists between migraine and insomnia that is unrelated to anxiety or depression, and insomnia is a risk factor for increased pain intensity, increased migraine impact, and chronicity in patients with migraine (6). EDS is associated with insomnia or other sleep disturbances in patients with migraine, and 10% of patients experience EDS during the premonitory, headache, or recovery phases of headache (5). RLS is a sensorimotor neurological disorder resulting in sleep onset or maintenance insomnia or daytime dysfunction. Meta-analyses have shown a high RLS comorbidity rate of 17% in migraine patients compared with 7% in nonmigraine individuals (11). According to a case–control study of migraine patients, patients with comorbid RLS had more severe depressive symptoms, greater daytime sleepiness, and headache-related disability than did those without RLS (12). Rapid eye movement sleep behavior disorder (RBD) is a parasomnia that causes sleep disruption and sleep-related trauma due to dream enactment in association with the loss of muscle atonia during REM sleep. Idiopathic RBD, which has been suggested to be associated with the risk of developing synucleinopathy, usually develops after the age of 50 years, while young-onset RBD has been associated with type 1 narcolepsy, depression and autoimmune disorders (13). According to a questionnaire-based study, dream-enacting behavior, suggestive of RBD, was more prevalent in patients with migraine than in healthy controls (24.2% vs. 14.3%) and was associated with headache-related disability (14). Independent studies have shown that coexisting sleep disorders such as insomnia, RLS, and daytime sleepiness decrease quality of life and increase the severity of pain in migraine patients (15). However, few studies have investigated the burden of multiple comorbid sleep disorders in patients with migraine.

Central sensitization (CS), the increased responsiveness of nociceptive neurons in the central nervous system to normal afferent input, is involved in cutaneous allodynia and migraine pathogenesis, such as trigeminal activation, cortical spreading depression, and headache chronification (16, 17). We previously reported the presence of RLS as a significant determinant of CS in migraine patients (18); thus, it is possible that the severity of CS may be related to the burden of sleep disorders.

Mediation models were designed to provide an explanation of how exposures cause presumed effects on outcomes and are useful for understanding the pathways and intermediates through which causal factors affect outcomes (19). In recent years, an increasing number of studies have used this analysis to evaluate direct and indirect factors involved in the relationship between two presumably related events, sleep and psychological stress (20, 21). In this study, we hypothesized that the burden of comorbid sleep problems would affect headache-related disability through the effects of CS and tested this hypothesis through mediation analysis.

## Methods

We performed a single-center cross-sectional study. This study was approved by the Institutional Review Board of Dokkyo Medical University Hospital. All participants provided written informed consent to participate in this study. A total of 215 consecutive patients with migraine (34 men/181 women, age 46.8 ± 11.7 years) from our headache outpatient clinic were included in this study after excluding 3 patients with missing data. Using G*power software (version 3.1.9.6), the sample size was calculated for ANCOVA of MIDAS and CSI scores in the five sleep problem groups by 15 covariates with an effect size of 0.25, an alpha error of 0.05, and a power of 0.8, resulting in a sample size requirement of 197 patients. This study included 215 patients with migraine, providing an adequate sample size.

Migraine was diagnosed by a headache specialist according to the International Classification of Headache Disorders, 3rd edition (ICHD-3) (22). Chronic migraine was defined as headache occurring at least 15 days per month for at least 3 months during which migraine features were evidenced for at least 8 days per month, and episodic migraine was defined as headache that occurred less than 15 days per month. Medication overuse headache (MOH) was diagnosed according to the ICHD-3 criteria (22).

We obtained information on smoking status, caffeine intake, alcohol intake, and body mass index from the questionnaires. Headache-related disability during the past 3 months was assessed with the Migraine Disability Assessment (MIDAS) questionnaire (score range, 0–270) (23). The Central Sensitization Inventory (CSI) has been widely used and validated for the assessment of symptoms related to CS (24). In this study, CS was assessed by the Japanese version of the CSI, which includes 25 items on physical symptoms related to CS (score range, 0–100) (25). RLS was diagnosed by a neurologist according to established criteria (26). The diagnosis of RLS requires four essential features: (1) an urge to move the legs, (2) the initiation or worsening of symptoms during periods of rest or inactivity, (3) partial or total relief of symptoms through movement, and (4) symptoms that occur only or are worse in the evening or night. Conditions that mimicked RLS were ruled out by neurological examination. A diagnosis of RLS was considered positive if the patient had lifetime symptoms. The Pittsburgh Sleep Quality Index (PSQI) is a widely used measure for sleep problems and is recommended for screening for insomnia (27). The PSQI consists of the following seven component scores (subscale score range, 0–3): C1, sleep quality; C2, sleep latency; C3, sleep duration; C4, habitual sleep efficiency; C5, sleep disturbance; C6, sleep medication use; and C7, daytime dysfunction. Insomnia was defined as a PSQI global score greater than 5 (28). Daytime sleepiness was measured using the Japanese version of the Epworth Sleepiness Scale (ESS); EDS was defined as an ESS score of 10 or greater (29). The Japanese version of the RBD screening questionnaire (RBDSQ-J) was used to assess symptoms of dream-enacting behavior, and probable RBD (pRBD) was defined as an RBDSQ-J score of 5 or greater (30). Suspected sleep apnea (SA) was defined as patients with snoring or sleep apnea witnessed at least 3 days per week. Depressive symptoms were assessed using the Beck Depression Inventory-II (BDI-II) (31).

## Statistical analysis

One-way analysis of variance (ANOVA) was used to analyze continuous variables, as appropriate, and chi-square tests were used to analyze categorical variables among groups. MIDAS or CSI scores in the five groups classified by the number of sleep problems were analyzed using analysis of variance (ANOVA) with a *post hoc* Bonferroni correction for differences from the 0 sleep-problem group. Multivariate logistic regression analysis was performed to assess the associations of the number of comorbid sleep problems with MIDAS and CSI scores using age, sex, aura, MOH, disease duration, alcohol intake, smoking, caffeine intake, comorbidities, episodic or chronic migraine, and the BDI-II score as adjustment factors. Mediation analysis was also conducted to assess whether CS severity mediates the association between the number of sleep disorders and the MIDAS score based on Baron and Kenny’s criteria (32). In the mediation analysis, the total effect of the number of sleep problems on the MIDAS score and the indirect effect mediated by the CSI total score were analyzed after adjustment for age, sex, aura, MOH, disease duration, alcohol intake, smoking, caffeine intake, comorbidities, episodic or chronic migraine, and the BDI-II score. The Sobel test was calculated by an interactive calculation tool for mediation tests.^1^

A two-sided *p* < 0.05 was considered to indicate statistical significance. IBM SPSS Statistics version 29 (IBM SPSS, Tokyo, Japan) was used for all the statistical analyses. GraphPad Prism for Mac (version 8; GraphPad Software, San Diego, United States) and Microsoft Excel version 16.18 were used to create the figures.

## Results

The characteristics of the patients with migraine are shown in Table 1. Overall, 24.2% of the patients had migraine with aura, 22.3% had MOH, and 24.2% had chronic migraine. The mean MIDAS score was 20.8 ± 33.6, and the mean CSI score was 32.3 ± 13.6. The mean PSQI global score and total ESS score were 8.0 ± 3.7 and 8.2 ± 4.8, respectively. RLS, insomnia, EDS, SA and pRBD were observed in 25.6%, 71.6%, 34.4%, 10.2%, and 21.4%, respectively, of the patients. At least one sleep problem was present in 87.0% of the patients. Table 2 shows the patient characteristics according to the number of comorbid sleep disorders. The caffeine intake rate; MIDAS, CSI, and BDI-II scores; and comorbidity rates of insomnia, EDS and pRBD were significantly different among the four groups. Insomnia was found in 89.1% of patients with two sleep problems, 93.3% of patients with three sleep problems, and 100% of patients with four or more sleep problems. The comorbidity rate of SA tended to increase as the number of sleep problems increased. The relationship between the number of sleep problems and the use of migraine prophylaxis was not clear.

**Table 1:** Characteristics of patients with migraine.

	Patients with migraine
*n* (M/F)	215 (24/181)
Age (y)	46.8 ± 11.7
Aura, *n* (%)	52 (24.2)
MOH, *n* (%)	48 (22.3)
NSAIDs	34 (70.8)
Triptans	14 (29.2)
EM/CM	163/52
Disease duration (y)	28.2 ± 12.5
Caffeine intake, *n* (%)	192 (89.3)
Alcohol intake, *n* (%)	93 (43.3)
Smoking, *n* (%)	29 (13.5)
Comorbidities, *n* (%)	119 (55.3)
Migraine prophylaxis, *n* (%)	92 (42.8)
CGRP mAbs	41 (19.1)
Antidepressants	35 (16.3)
Antiepileptics	22 (10.2)
MIDAS total score	20.8 ± 33.6
CSI score	32.3 ± 13.6
BDI-II score	13.3 ± 10.0
**Sleep problems**	
RLS, *n* (%)	55 (25.6)
Insomnia, *n* (%)	154 (71.6)
EDS, *n* (%)	74 (34.4)
SA, *n* (%)	22 (10.2)
pRBD, *n* (%)	46 (21.4)
**Number of sleep problems, *n* (%)**	
0	28 (13.0)
1	80 (37.2)
2	64 (29.8)
3	30 (14.0)
≥4	13 (6.0)

MOH, medication overuse headache; CM, chronic migraine; EM, episodic migraine; MIDAS, Migraine Disability Assessment; CSI, central sensitization inventory; BDI-II, Beck Depression Inventory-II; RLS, restless legs syndrome; EDS, excessive daytime sleepiness; SA, suspected sleep apnea; pRBD, probable rapid eye movement sleep behavior disorder; NSAIDs, nonsteroidal anti-inflammatory drugs; CGRP mAbs, calcitonin gene-related peptide monoclonal antibodies.

**Table 2:** Patient characteristics classified by the number of comorbid sleep problems.

	Number of comorbid sleep problems					
	0	1	2	3	≥4	*p* value
*n* (M/F)	28 (5/23)	80 (14/66)	64 (9/55)	30 (5/25)	13 (1/12)	0.900
Age (y)	50.8 ± 12.0	46.4 ± 12.0	45.6 ± 12.3	48.1 ± 9.2	43.7 ± 10.6	0.266
Aura, *n* (%)	5 (17.9)	20 (25.0)	13 (20.3)	12 (40.0)	2 (15.4)	0.214
MOH, *n* (%)	8 (28.6)	14 (17.5)	16 (25.0)	8 (26.7)	2 (15.4)	0.617
NSAIDs	5 (62.5)	11 (78.6)	12 (68.8)	5 (62.5)	2 (100.0)	0.772
Triptans	3 (37.5)	3 (21.4)	5 (31.3)	3 (37.5)	0 (0.0)	
EM/CM	20/8	65/15	47/17	21/9	10/3	0.673
Disease duration (y)	31.6 ± 11.6	28.2 ± 12.5	26.0 ± 13.0	29.3 ± 11.6	29.1 ± 12.6	0.367
Caffeine intake, *n* (%)	28 (100.0)	73 (91.3)	55 (85.9)	27 (90.0)	9 (69.2)	0.042
Alcohol intake, *n* (%)	13 (46.4)	34 (42.5)	26 (40.6)	16 (53.3)	4 (30.8)	0.666
Smoking, *n* (%)	1 (3.6)	9 (11.3)	9 (14.1)	8 (26.7)	2 (15.4)	0.124
Comorbidities, *n* (%)	13 (46.4)	43 (53.8)	33 (51.6)	22 (73.3)	8 (61.5)	0.241
Migraine prophylaxis, *n* (%)	12 (42.9)	34 (42.5)	27 (42.2)	15 (50.0)	4 (30.8)	0.841
CGRP mAbs	6 (21.4)	18 (22.5)	11 (17.2)	5 (16.7)	1 (7.7)	0.725
Antidepressants	6 (21.4)	15 (18.8)	7 (10.9)	6 (20.0)	1 (7.7)	0.517
Antiepileptics	4 (14.3)	12 (15.0)	3 (4.7)	2 (6.7)	1 (7.7)	0.274
MIDAS total score	9.2 ± 11.2	13.0 ± 28.8	22.9 ± 28.4	33.1 ± 52.5	55.9 ± 31.9	<0.001
CSI score	21.6 ± 9.8	28.1 ± 11.2	33.6 ± 10.9	44.5 ± 12.8	45.9 ± 15.8	<0.001
BDI-II score	6.7 ± 3.9	11.1 ± 9.3	14 ± 8.9	19.4 ± 10.4	23.5 ± 12.8	<0.001
**Sleep problems**						
RLS, *n* (%)	0 (0.0)	2 (2.5)	23 (35.9)	19 (63.3)	11 (84.6)	<0.001
Insomnia, *n* (%)	0 (0.0)	56 (70.0)	57 (89.1)	28 (93.3)	13 (100.0)	<0.001
EDS, *n* (%)	0 (0.0)	13 (16.3)	24 (37.5)	24 (80.0)	13 (100.0)	<0.001
SA, *n* (%)	0 (0.0)	7 (8.8)	6 (9.4)	24 (20.0)	3 (23.1)	0.064
pRBD, *n* (%)	0 (0.0)	2 (2.5)	18 (28.1)	13 (43.3)	13 (100.0)	<0.001

MOH, medication overuse headache; CM, chronic migraine; EM, episodic migraine; MIDAS, Migraine Disability Assessment; CSI, central sensitization inventory; BDI-II, Beck Depression Inventory-II; RLS, restless legs syndrome; EDS, excessive daytime sleepiness; pRBD, probable rapid eye movement sleep behavior disorder.

**Figure 1:**
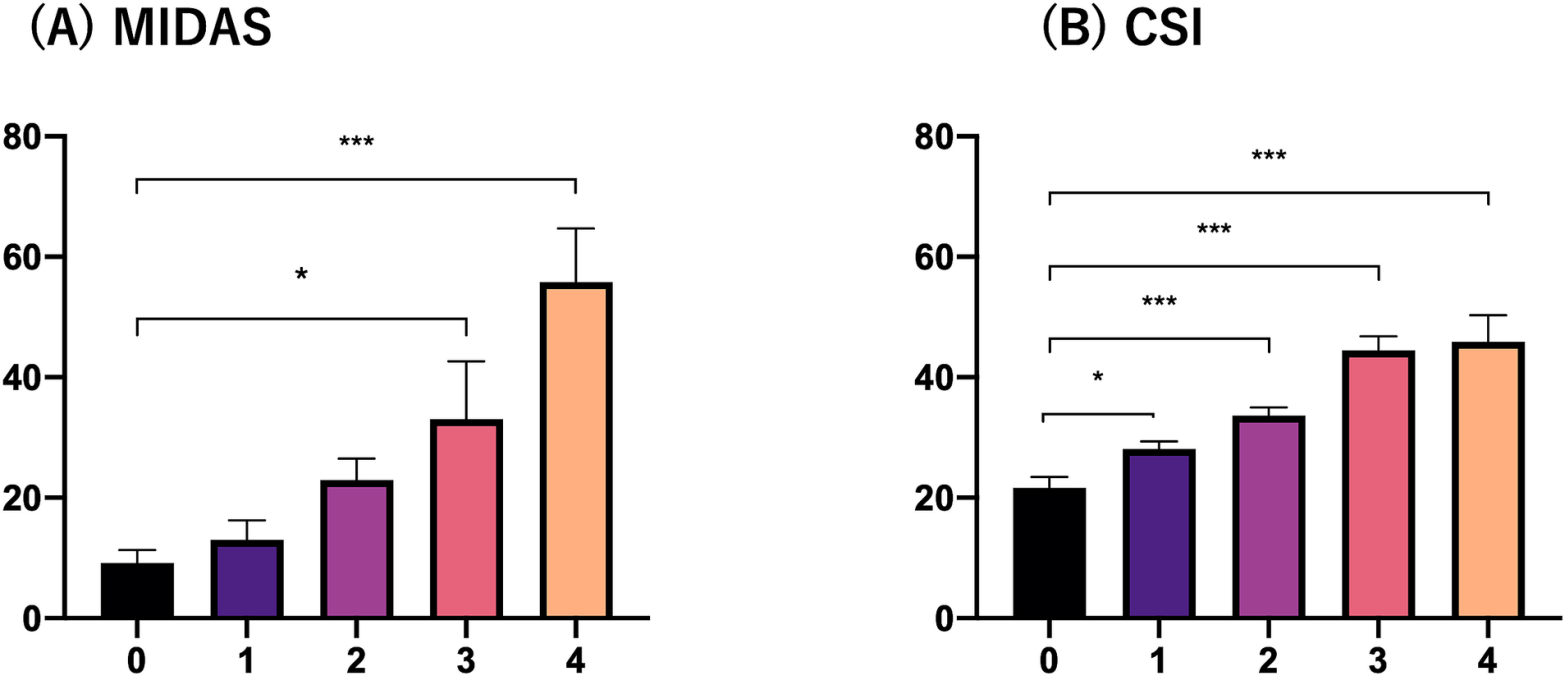
MIDAS and CSI scores according to the number of comorbid sleep problems. **(A)** MIDAS and **(B)** CSI. The vertical axis indicates the MIDAS or CSI score and the horizontal axis indicates the number of sleep problems.

**Figure 2:**
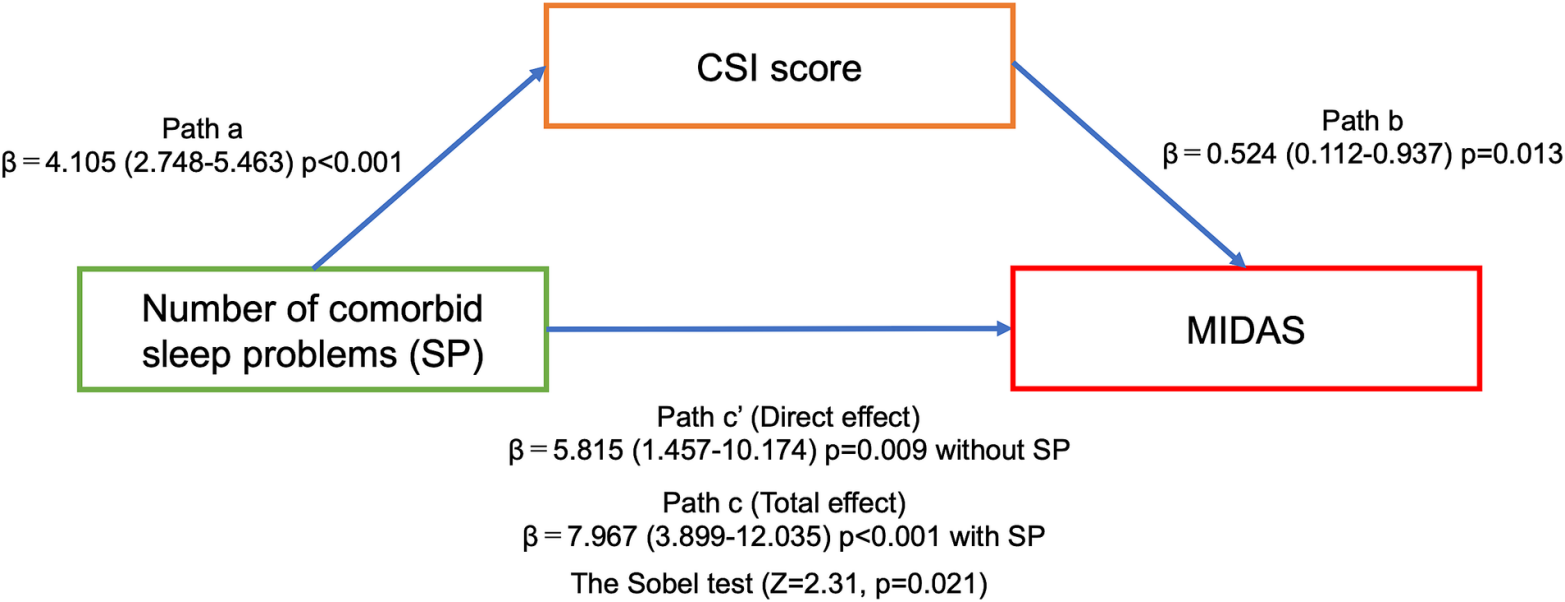
The association between the number of comorbid sleep problems and the MIDAS score mediated by the CSI score in mediation analysis. Path A represents the stratification analyses of the association between an increased number of sleep problems and the CSI score. Path B represents the CSI score as a mediator of the relationship between an increased number of sleep problems and the MIDAS score. Paths C and C′ represent the total and direct effects, respectively. All associations were adjusted for age, sex, aura, MOH, disease duration, alcohol intake, smoking, caffeine intake, comorbidities, episodic or chronic migraine, and the BDI-II score.

Figure 1 shows that the MIDAS and CSI scores were significantly higher in the three and four comorbid sleep problem groups than in the zero, one and two comorbid sleep problem groups. Multinomial logistic regression analysis controlling for adjustment factors showed that MIDAS scores increased significantly when three or more comorbid sleep problems were present compared to no sleep problem as a reference (Table 3). In contrast, CSI scores increased significantly when one or more sleep problems cooccurred compared with no sleep problem as a reference (Table 3). Mediation analysis revealed that an increased number of sleep problems had a direct effect on the increase in MIDAS score [β = 5.815 (95% CI, 1.457–10.174), *p* = 0.009] after adjustment for age, sex, aura, MOH, disease duration, alcohol intake, smoking, caffeine intake, comorbidities, episodic or chronic migraine and the BDI-II score. There was an association between the number of comorbid sleep problems [β = 4.105 (95% CI, 2.748–5.463), *p* < 0.001] and the CSI score, and the indirect effect was mediated by the CSI score [β = 0.524 (95% CI, 0.112–0.937), *p* = 0.013]. Based on Baron and Kenny’s criteria, the association between the number of sleep problems and the MIDAS score was mediated by the CSI score, which was further supported by the Sobel test (*Z* = 2.31, *p* = 0.021) (Figure 2).

**Table 3:** Multinomial logistic regression analysis of the association of the number of comorbid sleep problems with the MIDAS and CSI scores.

	cOR	95% CI	*p* value	aOR	95% CI	*p* value
***MIDAS***						
**Number of comorbid sleep problems**						
0	1.0 (ref)			1.0 (ref)		
1	1.016	0.985–1.048	0.306	1.014	0.980–1.049	0.439
2	1.035	1.004–1.066	0.026	1.032	0.998–1.068	0.067
3	1.043	1.011–1.075	0.007	1.038	1.002–1.076	0.037
4	1.050	1.018–1.084	0.002	1.049	1.010–1.088	0.012
***CSI***						
**Number of comorbid sleep problems**						
0	1.0 (ref)			1.0 (ref)		
1	1.072	1.021–1.127	0.005	1.066	1.001–1.136	0.045
2	1.121	1.064–1.181	<0.001	1.111	1.040–1.187	0.002
3	1.199	1.130–1.273	<0.001	1.183	1.095–1.279	<0.001
4	1.208	1.130–1.293	<0.001	1.196	1.090–1.311	<0.001

MIDAS, Migraine Disability Assessment; CSI, Central Sensitization Inventory; cOR, crude odds ratio; aOR, odds ratio adjusted for age, sex, aura, MOH, disease duration, alcohol intake, smoking, caffeine intake, comorbidities, episodic or chronic migraine, and the BDI-II score; 95% CI, 95% confidence interval.

## Discussion

Our study showed that migraine patients had multiple overlapping sleep problems. Furthermore, the greater the number of sleep problems present, especially three or more comorbid sleep problems, the greater the CSI and MIDAS scores. Insomnia has the highest complication rate among the various sleep problems in this study, and we believe that it has the highest impact on the degree of headache-related disabilities. Importantly, the number of sleep problems was directly related to the MIDAS score, and mediation analysis showed that CS was indirectly involved in the association between the number of sleep problems and headache-related disability. A clear reciprocal relationship between sleep and migraine headaches has been recognized. Sleep disturbances not only induce acute migraine attacks but are also risk factors for the chronicity and increased severity of migraine (4). Sleep disturbances have been reported to be associated with worsening headache frequency and severity in patients with migraine (33, 34). We previously reported that RLS (12) and pRBD (14) had negative effects on headache-related disability in patients with migraine. Furthermore, we found that the presence or absence of RLS at baseline had an effect on headache-related disability at the 7-year follow-up (35). However, few studies have investigated the impact of the number of overlapping sleep disorders on the degree of headache-related disability. As interventions for sleep disturbances, such as cognitive behavioral therapy for insomnia, can reduce the monthly headache frequency (36), it may be important to address sleep disturbances in migraine patients when their headache symptoms worsen. Screening for other treatable sleep disorders, such as RLS and sleep apnea, may also be important.

Several studies suggest a relationship between sleep disturbances and increased perception of pain, suggesting CS involvement. In healthy individuals, total sleep deprivation causes hyperalgesia to mechanical stimuli (37). Moreover, CS was shown to be associated with insomnia severity in cancer survivors with chronic cancer pain (38). In patients with knee osteoarthritis or chronic low back pain, sleep disturbances were associated with increased CS levels (39, 40). In addition, the presence of insomnia in patients with painful temporomandibular disorders, known as CS-related diseases (25), was associated with severe CS symptoms (41). In patients with migraine, CS is associated with chronicity and severity of pain (17), cutaneous allodynia (42), and photophobia (43). Cutaneous allodynia and osmophobia are more frequently associated with chronic migraine than with episodic migraine (44). The threshold for sound aversion was found to be lower in migraine patients with allodynia than in those without allodynia (45). These findings suggest that CS may be involved in the increase in migraine severity associated with various hypersensitivity symptoms, such as photophobia, phonophobia, osmophobia and allodynia. The intensity of migraine headaches reportedly increases with the degree of light sensitivity, sound sensitivity, smell sensitivity, and nausea (46). Furthermore, it has been reported that the greater the number of overlapping photophobia, phonophobia, and osmophobia symptoms is, the greater the degree of headache-related disability (47).

Glutamate is the most widely distributed excitatory neurotransmitter in the central nervous system and is thought to play an important role in the pathophysiology of migraine. Glutamate binds to receptors with distinct structural and functional properties and exerts complex actions in the central nervous system, and the interactions of different glutamate receptors with nociceptive and antinociceptive actions may be relevant to the induction of CS (48). Thus, blood glutamate levels could be a marker for CS and were significantly greater in the interictal phase in patients with migraine than in controls (49). Additionally, glutamate is a wake-promoting neurotransmitter that acts during wakefulness, and its activity is inhibited during non-REM sleep (50). A study evaluating GABAergic and glutamatergic activation using transcranial magnetic stimulation showed increased activation of glutamatergic intracortical activity in patients with insomnia compared to controls (51). In a study using magnetic resonance spectroscopy, thalamic glutamate/glutamine levels were increased in patients with RLS compared to healthy individuals and were correlated with wake time during the sleep period (52). CGRP, for which monoclonal antibodies (mAbs) have recently become available for novel migraine treatment, contributes to the underlying pathophysiology of migraine. Elevated levels of CGRP in the spinal cord stimulate astrocytes and microglial cells to release inflammatory mediators known to promote and maintain a state of CS (53). The CGRP monoclonal antibodies used for preventive migraine treatment have been reported to prevent the activation and sensitization of central trigeminovascular neurons (54) and reduce photosensitivity (55). Interestingly, CGRP appeared to maintain wakefulness, regulate nocturnal sleep maintenance, and mediate sleep-specific circadian output in a Drosophila model (56). Therefore, our results showing that the number of sleep problems is related to the degree of headache-related disability via the CS may be influenced by the neuropeptides involved in migraine pathophysiology. Although this study did not find an association between the rate of CGRP mAb use and the number of sleep problems, prospective studies, including pre- and posttreatment evaluations, are needed to determine whether CGRP mAb use is effective in treating sleep problems.

Migraine and sleep disorders share common neurotransmitters in the brainstem and hypothalamus that are involved in both conditions. In the brainstem, the dopaminergic periaqueductal gray, serotonergic dorsal raphe, noradrenergic locus coeruleus, dopaminergic posterior hypothalamus, and orexinergic lateral hypothalamus are involved in the transmission and regulation of pain while stimulating arousal and regulating sleep–wake transitions (57). Along with migraine, RLS is thought to pathophysiologically involve the dopaminergic system (58). However, the dopaminergic link between these two conditions may not be simple, as dopamine antagonists alleviate migraine premonitory symptoms and induce RLS symptoms (59). In addition, glymphatic dysfunction has been proposed as a common underlying mechanism for sleep disorders and headaches. Sleep contributes to brain homeostasis and the excretion of waste products via the glymphatic system (60). It has been shown that sleep deprivation reduces glycogen breakdown, causing cortical spreading depression and eventually leading to the development of migraine (61).

A limitation of this study is the lack of healthy controls. Our study used face-to-face interviews and validated questionnaires to evaluate various sleep problems, but objective sleep tests, such as polysomnography or multiple sleep latency tests, were not performed. We included clinically relevant sleep problems in patients with migraine patients, such as insomnia, EDS, pRBD, suspected SA and RLS, according to our or other study results. In this study, RLS was defined as a lifetime rather than a past-year occurrence, which may have led to an increased prevalence of RLS compared to previous studies. Also, the number of pRBD patients may have been overestimated because of the questionnaire-based assessment. Further studies should use polysomnography and multiple sleep latency tests with standardized criteria for the selection of sleep disorders. The number of headache days per month and headache severity are important parameters in pre- and posttreatment evaluations. In this study, however, there was no treatment intervention, and our main objective was to evaluate the association between the number of sleep problems and the degree of headache-related disability in migraine patients. In this study, 76% of patients had episodic migraine, including those with fewer headache days and milder pain severity. Therefore, this study used the MIDAS as the main outcome; the MIDAS is a widely validated instrument that can sensitively assess relevant disability even in patients with a low number of monthly headache days (62).

In conclusion, this study revealed an association between the burden of comorbid sleep problems and the severity of headache-related disability. Moreover, CS was shown to partially mediate this association.

## Data availability statement

The raw data supporting the conclusions of this article will be made available by the authors, without undue reservation.

## Ethics statement

The studies involving humans were approved by the Institutional Review Board of the Dokkyo Medical University Hospital. The studies were conducted in accordance with the local legislation and institutional requirements. The participants provided their written informed consent to participate in this study.

## Author contributions

KS: Conceptualization, Data curation, Formal analysis, Investigation, Methodology, Project administration, Writing – original draft. SS: Data curation, Investigation, Methodology, Project administration, Writing – review & editing. YH: Conceptualization, Formal analysis, Investigation, Methodology, Project administration, Writing – review & editing. KF: Investigation, Methodology, Writing – review & editing. HF: Data curation, Investigation, Methodology, Writing – review & editing. HS: Investigation, Methodology, Writing – review & editing. MH: Investigation, Methodology, Writing – review & editing. GK: Conceptualization, Funding acquisition, Investigation, Methodology, Project administration, Writing – review & editing. KH: Conceptualization, Investigation, Methodology, Project administration, Supervision, Writing – review & editing.

## Footnotes

^1^https://quantpsy.org/sobel/sobel.htm
